# Translational Potential of Therapeutics Targeting Regulatory Myeloid Cells in Tuberculosis

**DOI:** 10.3389/fcimb.2018.00332

**Published:** 2018-09-21

**Authors:** Nelita du Plessis, Leigh A. Kotze, Vinzeigh Leukes, Gerhard Walzl

**Affiliations:** Division of Molecular Biology and Human Genetics, Faculty of Medicine and Health Sciences, DST-NRF Centre of Excellence for Biomedical Tuberculosis Research, South African Medical Research Council Centre for Tuberculosis Research, Stellenbosch University, Cape Town, South Africa

**Keywords:** regulatory myeloid cells, myeloid-derived suppressor cells, Mycobacterium tuberculosis, host-directed therapy, immunotherapy

## Abstract

Despite recent advances in tuberculosis (TB) drug development and availability, successful antibiotic treatment is challenged by the parallel development of antimicrobial resistance. As a result, new approaches toward improving TB treatment have been proposed in an attempt to reduce the high TB morbidity and mortality rates. Host-directed therapies (HDTs), designed to modulate host immune components, provide an alternative approach for improving treatment outcome in both non-communicable and infectious diseases. Many candidate immunotherapeutics, designed to target regulatory myeloid immune components in cancer, have so far proven to be of value as repurposed HDT in TB. Several of these studies do however lack detailed description of the mechanism or host pathway affected by TB HDT treatment. In this review, we present an argument for greater appreciation of the role of regulatory myeloid cells, such as myeloid-derived suppressor cells (MDSC), as potential targets for the development of candidate TB HDT compounds. We discuss the role of MDSC in the context of Mycobacterium tuberculosis infection and disease, focussing primarily on their specific cellular functions and highlight the impact of HDTs on MDSC frequency and function.

## Introduction

The global TB health concern is exacerbated by the emergence of drug-resistant *Mycobacterium tuberculosis (Mtb)* strains. Other considerations, such as the substantial economic burden imposed by the length of TB treatment and the associated drug toxicity, favor the development of novel TB drugs (Islam et al., 2017). Surprisingly, the current pipeline for the development of new antibiotic compounds against *Mtb* remains slim. TB therapeutic research is now focused on the establishment of novel treatment strategies, such as host-directed therapies (HDTs), as an adjunctive approach to the current treatment regimen. HDTs aimed at modulating host immune homeostasis to ensure eradication of the invading pathogen, whilst simultaneously limiting tissue pathology, appears most promising. Similar HDT approaches correcting aberrant host pathways by way of targeting immune checkpoints, have shown huge success in cancer treatment plans. While immunotherapeutics has placed much emphasis on active enhancement of adaptive immune cell function through direct targeting of T-cell checkpoints, myeloid cells have recently emerged as equally attractive immune targets (Burga et al., 2013). Regulatory myeloid cells, such as myeloid-derived suppressor cells (MDSC), constitute a key innate immune checkpoint that impedes protective immunity in cancer (Young et al., 1987; Gabrilovich and Nagaraj, 2009). Common signaling pathways and similarities in immune regulation in malignancy and infectious disease, support the idea that cancer immunotherapeutic discoveries, can guide TB HDT strategies focused on pharmacological modulation of regulatory myeloid cells. We discuss the unfavorable role of regulatory myeloid cells in oncology, efforts to target MDSC in cancer clinical trials, knowledge on their negative contribution to *Mtb* control and highlight TB HDT compounds with potential to manipulate MDSC.

## Regulatory myeloid cells in tuberculosis: myeloid-derived suppressor cells

While the role of immunosuppressive regulatory T-cells have been demonstrated (Singh et al., 2012; Larson et al., 2013), the involvement of regulatory myeloid cells in TB, is not yet fully appreciated. In this regard, one of the mechanisms accounting for inadequate T-cell responses, is through defective engagement of innate immunity (Daker et al., 2015). Therefore, identification of new targets that regulate innate immune cell function and promote optimal activity of protective anti-TB immune responses, are likely to contribute to development of effective HDT targets.

Myeloid cells are the first responders to *Mtb* challenge during pulmonary infection and are critically involved in the induction of adaptive immunity, containment of bacilli and orchestration of inflammation. The key contribution of innate immunity in the initiation and regulation of adaptive immunity has led to the design of immunotherapies modulating innate cells, aimed at controlling diseases such as cancer (Qin et al., 2015). While MDSC are considered crucial in curbing inflammation-induced pathology, chronic or excess inflammation results in accumulation of MDSC (Ostrand-Rosenberg and Sinha, 2009). Overabundant MDSC, in turn, produce inflammatory mediators which recruit additional MDSC, thereby exacerbating inflammation (Cheng et al., 2008; Sinha et al., 2008). MDSC have also gained attention in the TB field due to their host immunosuppressive potential and ability to harbor Mtb bacilli (Knaul et al., 2014). MDSC frequencies are significantly expanded in the blood of TB patients, but decrease in number following successful TB chemotherapy (du Plessis et al., 2013). Several lines of evidence demonstrate the detrimental effect of MDSC on anti-TB immunity, including T-cell activation, proliferation, trafficking, regulatory T-cell induction and T-cell cytokine responses (du Plessis et al., 2013; Obregón-Henao et al., 2013; Knaul et al., 2014; Daker et al., 2015). MDSC may also impair phagocyte responses through production of IL-10 and TGF-β, inhibiting DC and macrophage function, and polarizing these cells toward a Th2 phenotypic response, as shown in tumor biology (Knaul et al., 2014). Such impairments are likely to affect Mtb control mechanisms, as well as the initiation and maintenance of effective adaptive immunity. MDSC are not only capable of regulating the intensity of T-cell responses to particular antigens, but also determine the numbers and activity of other immuno-regulatory cells. Given this immuno-modulatory capacity, MDSC should be considered as potential targets for fine-tuning the host response to *Mtb*. The major value of MDSC-immunotherapeutic strategies, is that these agents may be combined with traditional TB treatment and other HDT options to improve and optimize pathogen clearance.

## Pharmacological targeting of regulatory myeloid cells

The phenotypic and functional diversity of cellular subsets present within the myeloid compartment remains underappreciated and poorly investigated in the context of TB. The complexity of innate phagocytes in the lungs of TB patients is particularly striking, suggesting that detailed characterization is imperative to understanding the mechanisms of TB susceptibility or protection (Silver et al., 2016). MDSC, purposed to regulate inflammation, have gained attention due to their central role in prevention of host anti-tumor immunity and subsequent immune escape (Lesokhin et al., 2012). MDSC not only support tumor cell metastasis, proliferation and angiogenesis, but also create an immunosuppressive environment for cancer cell evasion of host immunity and chemotherapy-induced senescence, thereby promoting disease and treatment resistance (Gabrilovich et al., 2012; Bronte et al., 2016; Kumar et al., 2016). An area of intense research in the oncology field, is the identification and targeting of MDSC mechanisms and molecules supporting tumor escape. Reversal of MDSC function, inhibition of MDSC recruitment or depletion of MDSC numbers, have all shown promise in enhancing the activity of cancer vaccines and therapies in preclinical models, with growing evidence from clinical trials (Di Mitri et al., 2015; Draghiciu et al., 2015a).

## Strategies for reversing MDSC impact on anti-TB immunity

**Figure 1:**
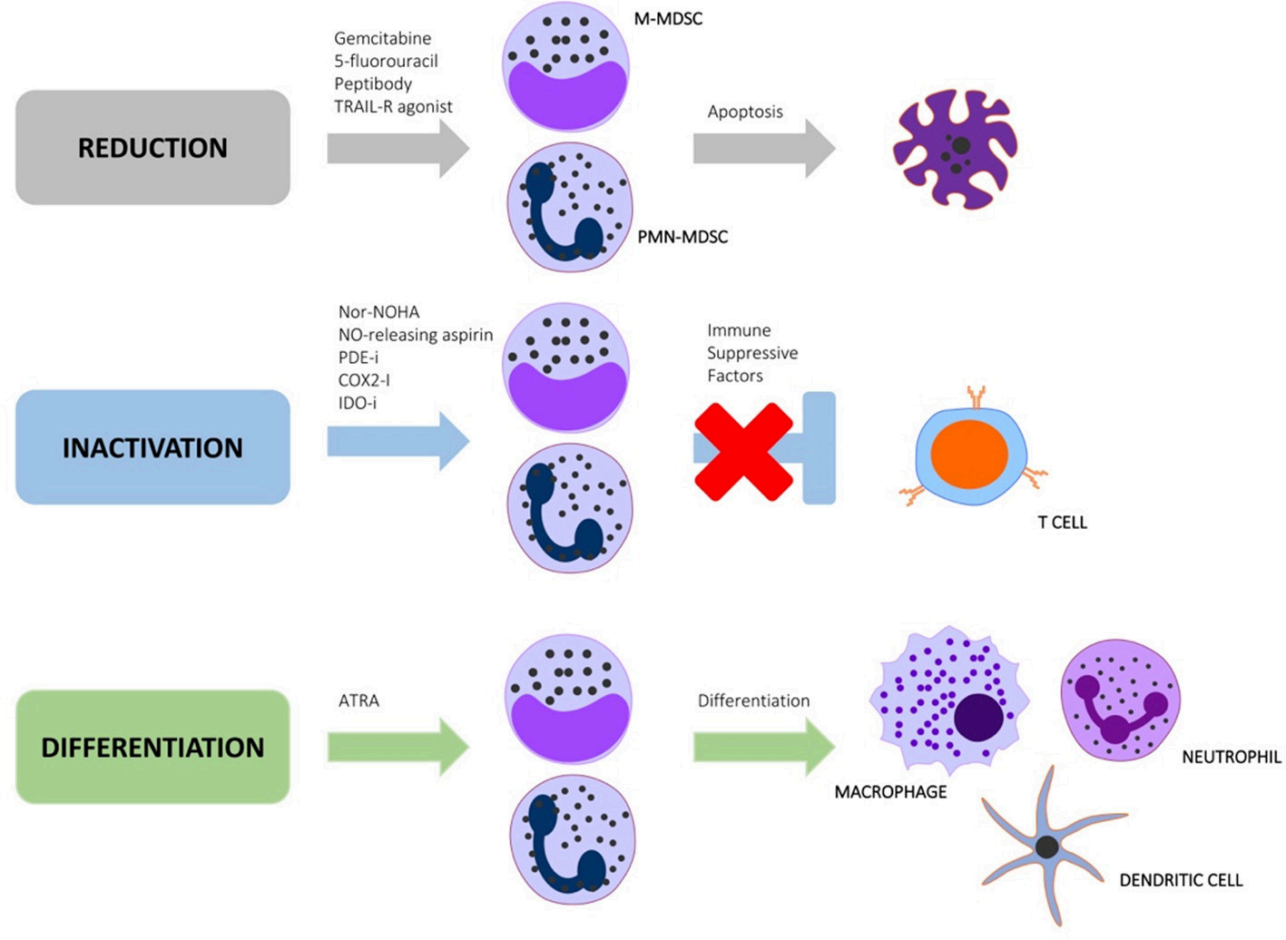
Immunotherapeutic strategies aimed at targeting regulatory myeloid cell pathways to reduce, inactivate or differentiate MDSC.

Regulatory myeloid cells such as MDSC have been successfully depleted with anti-Gr1+ antibodies in murine cancer models, with an associated reduction in tumor burden (Condamine et al., 2014). The Gr1+ antigen is, however, not present in humans, and is also a non-specific granulocyte marker, making this MDSC depletion strategy highly contentious (Xing et al., 2016). These findings do, however, suggest that depletion of MDSC is an effective immunotherapeutic approach. Other MDSC depletion strategies have shown greater success as cancer immunotherapy at pre-clinical and clinical trial level, and should be considered as repurposed treatment options for TB (Draghiciu et al., 2015a). MDSC targeting strategies can be categorized as approaches to (1) block MDSC inducing factors, (2) reverse MDSC functionality, and (3) differentiate MDSC into non-suppressive cells (Figure 1 and Table 1). There is, however, considerable cross-talk and overlap between these pathways, with numerous feedback mechanisms necessitating investigations in each stage of the TB disease spectrum.

**Table 1:** Agents affecting regulatory myeloid cell pathways have been tested as immunotherapeutics in cancer, some of which have also shown promise when evaluated in TB.

**Host-directed therapy**	**Regulatory myeloid response to host-directed therapy in cancer**	**References**	**Host-directed therapy in TB?**	**References**
**(1) INHIBITION OF MYELOID-DERIVED SUPPRESSOR CELL EXPANSION AND RECRUITMENT**				
Anti-IL-6R	Reduction of both granulocytic and monocytic MDSC subsets, reduction in tumor growth and improved T cell functions. **Murine model**.	Sumida et al., 2012	Experimental stages—effect on MDSC, in the context of TB, yet to be evaluated. Troublesome results being found in *M.tb* infection model where blockade of IL-6R results in an increase in susceptibility to infection in mice. **Murine infection model**.	Okada et al., 2011
Etanercept Anti-TNF-α	Reduced MDSC frequencies in the blood with simultaneous delayed tumor growth and volume. Potentially a CD8 T cell-dependent mechanism. **Murine and human model**.	Bayne et al., 2012; Atretkhany et al., 2016	Experimental stages—effect on MDSC, in the context of TB, yet to be evaluated. Troublesome risk of anti-TNF-α treatment resulting in reactivation of active disease, especially in latent infection cases. **Human infection model**.	Wallis, 2009
Anti-GM-CSF	Variable results. These included the impairment of GM-CSF-mediated MDSC differentiation in the supernatant of cancerous lesions following treatment with neutralizing anti-GM-CSF antibodies, as well as reduced MDSC accumulation in the spleen following GM-CSF knockout. **Murine model**.	Dolcetti et al., 2010; Ma et al., 2017	Experimental stages—effect on MDSC, in the context of TB, yet to be evaluated. GM-CSF appears to confer a protective role in TB owing to its activation of macrophages to inhibit intracellular *M.tb* growth, therefore, a GM-CSF targeted therapy may prove detrimental to the host. **Murine and human infection model**.	Robinson, 2017; Rothchild et al., 2017
Anti-VEGF	Reduced numbers of circulating VEGFR1-expressing MDSC which may restore immunocompetency. **Murine and human model**.	Kusmartsev et al., 2008	Experimental stages—effect on MDSC, in the context of TB, yet to be evaluated. In a TB model, anti-VEGF treatment promotes vascular normalization, reduces hypoxic areas within the TB granuloma and thereby provide improved delivery mechanisms for current anti-Tuberculosis therapies. **Murine and human infection model**.	Datta et al., 2015
Anti-IL-17R + Anti-IFN-γR	Reduced MDSC numbers, increased number of T cells, and reduced tumor development. **Murine model**.	He et al., 2010	Experimental stages. Anti-IL-17R has been shown to reduce the granulocytic subset of MDSC in a murine TB model. **Murine infection model**.	Freches et al., 2013; Lombard et al., 2016; Segueni et al., 2016
Sunitinib Tyrosine kinase inhibitor (multitargeted)	Inhibition of STAT3 through the inhibition of the Jak/Stat pathway reverses MDSC expansion. **Murine and human model**.	Chen et al., 2015a; Draghiciu et al., 2015b,c; Ko et al., 2009; Xin et al., 2009	Effect on MDSC, in the context of TB, yet to be evaluated.	N/A.
Gefitinib Tyrosine kinase inhibitor (targets EGFR mutations)	S100A9^+^ MDSC-derived macrophages in the tumor microenvironment mediate resistance to tyrosine kinase inhibitors targeting EGFR mutations. **Human model**.	Maemondo et al., 2010; Feng et al., 2018	Experimental stages—effect on MDSC, in the context of TB, yet to be evaluated. Gefitinib has been shown to inhibit STAT3 which is crucial for the expansion of MDSC, making it a promising target for directed therapies. **Murine and human infection model**.	Stanley et al., 2014; Sogi et al., 2017
Imatinib Tyrosine kinase inhibitor (targets ABL family)	Reduces the number of MDSC, as well as the levels of arginase 1, to those of healthy control patients. **Human model**.	Giallongo et al., 2014	Experimental stages—effect on MDSC, in the context of TB, yet to be evaluated. Imatinib reduced the number of granulomatous lesions and bacterial load in a murine TB model. **Murine and human model**.	Napier et al., 2011; Kalman et al., 2017
5-Fluorouracil (5-FU) Antimetabolite – Thymidylate synthase inhibitor	Reduces the number of MDSC without affecting other lymphocyte and myeloid population frequencies, except those of B cell population frequencies which are increased. **Murine model**.	Vincent et al., 2010	Experimental stages—effect on MDSC, in the context of TB, yet to be evaluated. Potential mycobacterial resistance threatens further investigation of this therapy in the context of TB. ***in vitro*** **infection model**.	Singh et al., 2015
Gemcitabine Antimetabolite – DNA synthesis inhibitor		Suzuki et al., 2005; Le et al., 2009	Effect on MDSC, in the context of TB, yet to be evaluated. Potentially increases the risk of reactivation of *M.tb* infection or increases susceptibility to *M.tb* infection in cancer patients.	Not Applicable.
MiR-155 Inhibitor	Reduces the expansion of the MDSC population within tumor-bearing mice and tumor growth. **Murine and human mode**.	Fabani et al., 2010; Li et al., 2014	Experimental stages—effect on MDSC, in the context of TB, yet to be evaluated. Inconclusive evidence exists that MiR-155 is indeed disease-promoting and needs to be investigated further. **Murine and human infection model**.	Huang et al., 2015; Iwai et al., 2015; Wagh et al., 2017; Etna et al., 2018
MiR-21 Inhibitor		Li et al., 2014; Chen et al., 2015b; Drakaki et al., 2017	Experimental stages—effect on MDSC, in the context of TB, yet to be evaluated. **Murine infection model**.	Wu et al., 2012
**(2) INHIBITION OF MYELOID-DERIVED SUPPRESSOR CELL FUNCTION**				
Nor-NOHA Arginase-1 Inhibitor	Delays tumor growth by reversing MDSC function. **Murine model**.	Rodriguez et al., 2003, 2004	Experimental stages. Nor-NOHA inhibits ARG1 in phagocytes, resulting in the reduction of mycobacterial growth and lowering of IL-10 production. **Murine and human infection model**.	Talaue et al., 2006; de Oliveira Fulco et al., 2014; Mason et al., 2015
NO-aspirin NOS and COX-2 Inhibitors	Reverse MDSC-mediated immunosuppression in both *in vitro* and *in vivo* cancer models, with marked reductions in MDSC frequencies and delayed tumor growth. **Murine and human model**.	Wu and Morris, 1998; Fiorucci et al., 2000; Bronte and Zanovello, 2005; Corazzi et al., 2005; Molon et al., 2011	NO-aspirin yet to be tested. Conflicting aspirin data. NO inhibitor findings contradict NO-aspirin data as NO inhibitors resulted in heightened bacterial burdens, increased lung pathology and reactivation of latent infection. **Murine infection model**.	Chan et al., 1995; Botha and Ryffel, 2002; Byrne et al., 2007
IDO Inhibitors	Successful inhibition of MDSC expansion in tumors and reduced immunosuppressive effects. **Human model**.	Wang et al., 2014; Holmgaard et al., 2015	Experimental stages. Effect of IDO-i have yet to be evaluated. Blocking of IDO does, however, reduce clinical manifestations of TB and alter granuloma organization. **Murine, macaque and human infection model**.	Hirsch et al., 2016; Gautam et al., 2018
PDE-5 Inhibitors Sildenafil and Tadalafil	Reversal of MDSC functions and augmentation of anti-tumor immunity via the inhibition of the degradation of cyclic guanosine monophosphate (cGMP). This results in a reduction of ARG1 and iNOS expression. **Murine and human model**.	Serafini et al., 2006; Noonan et al., 2014	Experimental stages—effect on MDSC, in the context of TB, yet to be evaluated. **Murine and Rabbit infection model**.	Subbian et al., 2011, 2016; Maiga et al., 2013, 2015
COX2 Inhibitors Indomethacin and Etoricoxib	Downregulation of the production of ARG1 and iNOS by MDSC, resulting in the reduction of suppressive MDSC functions. May also reduce MDSC numbers or block MDSC activation. **Murine model**.	Rodriguez et al., 2005; Veltman et al., 2010; Fujita et al., 2011	Experimental stages. COX2-i enhance Th1 immunity and downregulate the frequency of *M.tb*-induced Tregs, but their MDSC-specific effects have not yet been evaluated. **Murine and human infection model**.	Hernández-Pando et al., 2006; Tonby et al., 2016
PD-L1 Inhibitors	MDSC-mediated immunosuppression is abrogated. **Murine and human model**.	Pilon-Thomas et al., 2010; Kleffel et al., 2015; Sharma and Allison, 2015b; Kleinovink et al., 2017	Experimental stages—effect on MDSC, in the context of TB, yet to be evaluated. Promising results have been shown in a TB model with the restoration of T cell responsiveness, cytokine secretion and proliferation, however immune reactivation responses are troublesome. **Human infection model**.	Hassan et al., 2015; Reungwetwattana and Adjei, 2016
Calprotectin (S100A8/9) Inhibitor	Inhibition of MDSC function resulting in reduced tumor growth. **Murine model**.	Sinha et al., 2008	Experimental stages—effect on MDSC, in the context of TB, yet to be evaluated. Upregulation of calprotectin in serum of TB patients is known to correlate with disease severity and pathology. Calprotectin is, therefore, a promising target for directed therapeutics. **Murine infection models**.	Kang et al., 2011; Gopal et al., 2013
**(3) MATURATION/DIFFERENTIATION OF MYELOID-DERIVED SUPPRESSOR CELLS INTO NON-SUPPRESSIVE CELLS**				
ATRA Retinoid-activated transcriptional regulator activator	Maturation of early myeloid cells into fully differentiated, non-immunosuppressive cells via the upregulation of GSH which reverses MDSC suppressive functions. **Murine and human models**.	Kuwata et al., 2000; Gabrilovich et al., 2001; Luo and Ross, 2006; Nefedova et al., 2007; Nakanishi et al., 2008; Gabrilovich and Nagaraj, 2009	Experimental stages. Effect of ATRA on MDSC in a TB model has been somewhat successful with an observed restoration of T cell numbers, reduced bacterial burden and lung pathology. **Murine infection models**.	Knaul et al., 2014

## Inhibition of MDSC expansion and recruitment

### Cytokines

In cancer, mediators known to enhance the expansion of MDSC include interleukin-6 (IL-6), tumor necrosis alpha (TNF-α), granulocyte-macrophage colony-stimulating factor (GM-CSF), cyclooxygenase-2 (COX2), prostaglandins, stem-cell factor (SCF), macrophage colony-stimulating factor (M-CSF), vascular-endothelial growth factor (VEGF), IL-1β and IL-17 (Shipp et al., 2016). A recent study has shown that blocking of the IL-6 receptor (IL-6R) or TGF-β in tumor-bearing mice, decreases both monocytic and granulocytic MDSC subsets, alongside a reduction in tumor growth and improvement in T-cell function (Sumida et al., 2012). In contrast, results with GM-CSF treatment have been variable (Ma et al., 2017). Mice deficient in IL-17R and IFN-γR, also demonstrated lower MDSC levels and increased T-cells that were associated with a reduction in tumor development (He et al., 2010).

In TB, some of these cytokines are also being investigated as potential targets in HDT approaches, but none have investigated the effect on MDSC specifically. Instead, adjunctive cytokine treatment as intervention in TB has, however, largely focused on supplementation of mediators which activate macrophages to promote mycobacterial killing or blockade of pro-inflammatory cytokine signaling to limit lung damage (Condos et al., 1997, 2003; Vogt and Nathan, 2011). The majority of studies report on IFN-γ treatment of TB and demonstrate a reduction in pro-inflammatory cytokine production, enhancement of TB-specific CD4 T-cell responses, enhanced sputum conversion and reduced radiological involvement, although some inconsistent outcomes have been reported (Park et al., 2007; Dawson et al., 2009). IFN-γ treatment has also shown promise as HDT in a small study on Cuban patients infected with non-tuberculous mycobacterial lung disease, such as *M. avium* (Suárez-Méndez et al., 2004). This suggests that some HDTs may also be beneficial in the treatment of NTM. It was shown that IFN- γ improved the extent and clearance rate of pulmonary and radiological symptoms of these patients by the month 18 time point (Suárez-Méndez et al., 2004). Pre-clinical data on anti-TNF-α, anti-VEGF and IL-6R blockers as TB HDT in TB animal models and case studies of severe pulmonary TB patients have been encouraging (Okada et al., 2011; Datta et al., 2015). It remains crucial to appreciate the complex role of cytokines in TB immune regulation, necessitating in depth characterization of the optimal cytokine dose and timing to determine the cytokine's effect on MDSC induction, and resultantly on disease modulation.

### Enzymes and transcription factors

In cancer, several of the cytokine mediators targeted by TB HDTs, trigger activation of the transcription factor, signal transducer and activator of transcription 3 (STAT3), which activates the signaling pathway mediating tumor-MDSC induction (Gao et al., 2007). In cancer, STAT3 is critically involved in MDSC expansion by stimulating expression of immature myeloid cell (IMC) genes involved in MDSC development (Tartour et al., 2011; Sansone and Bromberg, 2012; Table 1, Figure 1). STAT3 is phosphorylated by the tyrosine kinase Janus kinase/signal transducer and activator of transcription (Jak/Stat) pathway (Nicolas et al., 2012). Agents targeting these kinases in cancer patients are being investigated in an attempt to reverse MDSC expansion. For example, the tyrosine kinase inhibitor (TKI) sunitinib, blocks MDSC expansion in cancer patients and tumor bearing mice (Chen et al., 2015a; Draghiciu et al., 2015b,c). Furthermore, STAT3 overexpression in myeloid cells trigger expansion of MDSC and upregulate S100A9, which directs MDSC accumulation (Cheng et al., 2008; Wu et al., 2011). In TB, protein kinase inhibitors have also emerged as attractive candidates in the development of antimicrobial drugs. The TKI, gefitinib, a FDA-approved compound for the treatment of non-small-cell lung carcinoma, has recently been tested in human TB due to its ability to inhibit the epidermal growth factor receptor (EGFR) and activate autophagy to restrict bacterial growth. Gefitinib demonstrated *in vitro* and *in vivo* efficacy against Mtb infection, although the involvement of MDSC has not been considered (Stanley et al., 2014; Sogi et al., 2017). The TKI, imatinib, has also shown therapeutic efficacy in TB mouse models, leading to the initiation of a pre-clinical study on Mtb infection in a non-human primate model, but again, the role of MDSC was not investigated (Napier et al., 2011; Kalman et al., 2017). Inhibitors of protein kinase R are also being screened in TB mouse models, whereas other anti-cancer kinase inhibitors such as sunitinib, malate and curcumin analogs with known effects on tumor-derived MDSC induction, remains to be tested in TB.

### Cytotoxic agents

In cancer, selected cytotoxic cancer agents have also been used to deplete MDSC through a yet undefined mechanism(s). For example, 5-fluorouracil (5-FU) and gemcitabine treatment reduces the number of MDSC without affecting the frequency of T-cells, dendritic cells, NKT- or NK-cells, yet increases the B-cell population (Le et al., 2009; Vincent et al., 2010). In TB, the effect of 5-FU on host immunity, more specifically, MDSC, has not been tested, and although its direct bactericidal action against Mtb has been reported, mycobacterial resistance to 5-FU continues to be a concern (Singh et al., 2015). Efforts evaluating 5-FU as TB HDT has been directed to investigation on inhibitors of phosphodiesterase-5 (PDE-5), which has a known effect on MDSC function (see section Phosphodiesterase Inhibitors).

### Micro-RNA

With antisense technology improving, targeting of microRNAs (MiR) by immunotherapeutics is increasingly considered (Li et al., 2014). In cancer, MiR-155 and MiR-21 are critically required for the expansion of MDSC in tumor-bearing mice and to facilitate tumor growth; miR 93-106b cluster regulate expression of PD-L1 on MDSC; while a specific role for miR-142-3p has recently been suggested in cancer through preventing differentiation of myeloid cells (Li et al., 2014; Chen et al., 2015b). The role of MiR in TB continues to be unraveled, as reports emerge of Mtb facilitating expression of MiR-155 to disrupt the process of autophagy (Wagh et al., 2017; Etna et al., 2018). Although MiR-155 is responsive to TB therapy, it remains inconclusive if its expression is disease promoting, as MiR-155 knockout mice are susceptible to Mtb infection (Iwai et al., 2015; Wagh et al., 2017). MiR-21 is also upregulated following Mtb infection, reportedly to escape the host immune response by downregulating the genes for TNF-α and IL-6 (Wu et al., 2012). Also in TB, HDT manipulation of other miR, controlling key myeloid functions, has also been proposed. Although not yet tested in clinical trials, Mtb induced expression of miR-142-3p targets an actin-binding protein leading to reduced phagocytosis in primary human macrophages (Bettencourt et al., 2013). MiR-106b-5p was also specifically upregulated in human macrophages following Mtb infection to reduce function by lowering cathepsinS expression and favoring Mtb survival (Pires et al., 2017). Greater understanding of MiR function and regulation, also in the context of regulatory myeloid cells is, however, required before therapeutic targeting of host MiR becomes a reality. This is likely to be accompanied by the requirement of cell-specific delivery techniques, to avoid potential off-target immunological effects (Iannaccone et al., 2014).

## Inhibition of MDSC function

### Enzyme inhibitors

#### Arginase-1

In cancer, a critical mechanism whereby MDSC induce lymphocyte suppression is by local depletion of essential amino acids required for T-cell proliferation. Tumor-MDSC highly express the enzyme arginase-1 (ARG1) which catabolizes l-arginine to urea and ornithine (Wu and Morris, 1998; Bogdan, 2001), thereby inhibiting T-cell proliferation through decreased CD3-theta chain expression (Rodriguez et al., 2003, 2004). In lung cancer, treatment with the arginase inhibitor N^ω^-hydroxy-L-arginine (nor-NOHA) delays tumor growth by reversing MDSC function (Rodriguez et al., 2004). **In TB**, Nor-NOHA inhibition of ARG1 in phagocytes has also shown promise, through its reduction of mycobacterial growth and lowering of IL-10 production *in vitro* (Talaue et al., 2006; de Oliveira Fulco et al., 2014).

#### iNOS

In cancer, MDSC also generate oxidative stress by increasing levels of reactive oxygen species (ROS) and inducible nitric oxide (iNOS) with resultant immunosuppressive effects (Bronte and Zanovello, 2005; Youn et al., 2008). ROS and iNOS activity also steers the production of harmful peroxynitrites, H202 and NO (Schmielau and Finn, 2001; Youn et al., 2008; Table 1, Figure 1). Although NO is crucial, in TB, to mycobacterial control, nitrogen and oxygen intermediates suppress T-cell function by nitration of the T-cell receptor (Nagaraj et al., 2007), induction of T-cell apoptosis (Mannick et al., 1999), reduction of MHC expression(Harari and Liao, 2004) and reduction of CD3-theta chain expression (Schmielau and Finn, 2001). NOS inhibitors have been shown to reverse MDSC-mediated immunosuppression in both *in vitro* and *in vivo* cancer models (Wu and Morris, 1998; Bronte and Zanovello, 2005). For example, nitro-aspirin (NO-aspirin), a new molecule in which aspirin is covalently linked to a NO-group, suppresses the production of ROS and provides feedback inhibition to iNOS (Fiorucci et al., 2000). *In vitro* and *in vivo* models of NO-aspirin treatment have also been shown to reduce MDSC numbers, reverse MDSC induced inhibition of T-cell responses by reducing CCL2 chemokine nitration and delay tumor growth (Molon et al., 2011). NO-aspirin also inhibits the COX-2 enzyme, another MDSC inducer (Corazzi et al., 2005). In TB, information on aspirin as HDT has been conflicting, but the effect of NO-aspirin remains to be investigated (Byrne et al., 2007). In spite of this, findings from murine TB emphasize NO as vital component in innate immune control of Mtb, and argues against the use of NO inhibitors as these result in heightened bacterial burden, increased lung pathology and mortality (Chan et al., 1995) and reactivation of latent Mtb infection (Botha and Ryffel, 2002).

#### Phosphodiesterase inhibitors

Phosphodiesterase-5 (PDE-5) inhibitors (PDE-5-i), such as sildenafil and tadalafil, have good safety profiles as these have been used for decades to treat pulmonary hypertension, cardiac hypertrophy and erectile dysfunction. PDE-5-i were shown to reverse MDSC function in cancer patients and augment anti-tumor immunity by inhibiting the degradation of cyclic guanosine monophosphate (cGMP), leading to reduction in ARG1 and iNOS expression (Serafini et al., 2006; Noonan et al., 2014). Promising pre-clinical findings demonstrate that treatment with PDE-5-i improve T-cell responses, delay tumor growth and abrogate Treg proliferation in several cancer types (Serafini et al., 2006). This has resulted in a number of clinical trials on PDE-5-i in cancer, including a study evaluating whether treatment of oropharyngeal carcinoma patients with tadalafil could enhance T-cell tumor infiltration (NCT00843635); whether tadalafil can improve responses to dexamethasone chemotherapy (NCT01374217), if sildenafil treatment improves the outcome of non-small cell lung carcinoma (NCT00752115), or if tadalafil in combination with a novel vaccine and gemcitabine chemotherapy or radiation therapy, improves cancer outcome (NCT01342224).

In TB, the severe side effects associated with thalidomide, has led to the consideration of analogs, such as PDE-i, with similar potential to inhibit TNF-α (Aragon-Ching et al., 2007). PDE-i have been shown to decrease TB disease severity, reduce lung pathology and bacillary load in mouse models (Subbian et al., 2011, 2016; Maiga et al., 2013, 2015). The effect of PDE-i on immune cell phenotypes and function during human Mtb infection and TB disease remains poorly defined, but future and ongoing trials could cast more light on the impact of PDE-i on TB host immune responses, and necessitate evaluation of the effect on MDSC (NCT02968927).

#### Cyclooxygenase inhibitors

Prostaglandin-E2 (PGE-2) has both pro-inflammatory and immune-suppressive properties and is synthesized by cyclo-oxygenase-2 (COX2). MDSC highly express the PGE-2 receptor, E-prostanoid 4, which, upon binding, induces ARG1 (Rodriguez et al., 2005). In cancer, Treatment of MDSC with COX inhibitors (COX2-i) have shown to down-regulate their production of ARG1 and iNOS, thereby reducing MDSC suppressive function (Veltman et al., 2010). COX2-i may thus target MDSC on multiple levels, either by reducing their numbers or by blocking their activation (Rodriguez et al., 2005; Fujita et al., 2011). In TB, COX2-i also significantly improved host immunity to Mtb in animal models by enhancing Th1 immunity (Hernández-Pando et al., 2006). *In vitro* treatment of TB patients' blood samples with the COX2-i, indomethacin, a nonsteroidal anti-inflammatory drug, downregulated the frequency of Mtb-induced Tregs and impaired T-cell proliferation and antigen-specific cytokine responses (Tonby et al., 2016). Although this study did not consider the effect on regulatory myeloid cells, an ongoing trial evaluating the impact of the COX2-i, etoricoxib, on immune-mediated host clearance of Mtb, will allow for assessment of MDSC functionality (NCT02503839).

#### Indoleamine 2,3-dioxygenase inhibitors

Monocytic MDSC also express high levels of the enzyme indoleamine 2,3-dioxygenase (IDO) that mediates immunosuppression through a mechanism involving regulatory T-cells (Tregs) (Holmgaard et al., 2015). In cancer, inhibition of IDO successfully blocks expansion of MDSC in tumors, as well as reducing the immunosuppressive effects of MDSC in IDO deficient mice (Yang, 2009; Wang et al., 2014; Holmgaard et al., 2015). Mtb is also a potent inducer of IDO (Hirsch et al., 2016). In TB, even though the mycobacterial burden in IDO-deficient mice is comparable to those of wild type mice (Blumenthal et al., 2012), a recent report demonstrated that blocking of IDO decreases both the clinical manifestations of TB as well as microbial and pathological correlates in macaques by altering granuloma organization (Gautam et al., 2018). Additionally, IDO inhibitors may reduce the number of MDSC in the lungs of TB patients, however this remains to be tested.

### Checkpoint inhibitors

In cancer, tumor-derived MDSC highly express programmed death ligand-1 (PD-L1), which engages the PD-1 receptor on T-cells, resulting in an exhausted phenotype (Jiang et al., 2015; O'Donnell et al., 2017). Blocking of PD-L1 abrogated MDSC-induced immune suppression in a murine melanoma model (Kleffel et al., 2015; Kleinovink et al., 2017).

In TB, the use of a PD-L1 checkpoint inhibitor has shown some promise in an *in vitro* model through its restoration of T-cell responsiveness to TB antigens, cytokine secretion and proliferation (Sharma and Allison, 2015a). Similarly, TB treatment has been shown to reduce expression of the genes responsible for PD-L1 expression on T-cells and natural killer cells (Hassan et al., 2015). However, by reactivating the immune system through treatment with this checkpoint inhibitor, two cancer patients have developed active TB disease (Reungwetwattana and Adjei, 2016). This highlights the complexities associated with modulation of immune regulatory molecules, such as PD-L1, which are upregulated on multiple immune cell subsets in a range of disease conditions (Shen et al., 2016). Even so, inhibition of PD-L1 on immune regulatory cells such as MDSC in TB, merits further investigations.

In cancer, the calcium-binding pro-inflammatory alarmin, calprotectin (S100A8/9), is highly expressed on murine tumor-derived MDSC and contributes to the induction of MDSC (Sinha et al., 2008). Interference with S100A8/A9 signaling inhibits MDSC function, leading to decreased tumor growth (Sinha et al., 2008). In TB patients, serum abundance of S100A8/9 correlates with disease severity while also mediating neutrophilic inflammation and lung pathology in Mtb-infected experimental animals (Kang et al., 2011; Gopal et al., 2013). Given the association of S100A8/9 with MDSC, current TB treatment strategies and lung inflammation may thus benefit from targeting this molecule, with the expectation that it could limit immunosuppression and neutrophilic influx.

## Maturation/differentiation of MDSC in non-suppressive cells

Differentiation of MDSC into mature myeloid cells without immunosuppressive functionalities is another promising immunotherapeutic strategy. Various studies have shown enhanced MDSC levels in the bone marrow and spleen of vitamin-A deficient mice (Kuwata et al., 2000; Walkley et al., 2002).

### All-trans retinoic acid

All-trans retinoic acid (ATRA) is a vitamin-A metabolite that activates retinoid-activated transcriptional regulators (Nakanishi et al., 2008). These factors activate target genes resulting in the maturation of early tumor-associated myeloid cells into fully differentiated, non-immunosuppressive cells (Nefedova et al., 2007). In cancer, ATRA treatment also up-regulates glutathione (GSH), a ROS scavenger, thereby reversing MDSC immunosuppressive function (Nefedova et al., 2007). Furthermore, ATRA treatment of MDSC led to their differentiation into mature DC, granulocytes and monocytes, through their upregulation of differentiation markers such as HLA-DR (Lathers et al., 2004; Gabrilovich and Nagaraj, 2009). On the other hand, ATRA also increases expression of the transcription factor FoxP3, thereby inducing the development of Tregs which are considered to be detrimental to anti-tumor and anti-TB immunity alike (Ma et al., 2014). Despite this potential deleterious side effect, several clinical trials are evaluating ATRA as treatment option to modulate MDSC in cancer patients (NCT00601796; NCT00618891).

In TB, ATRA and other retinoic acids have shown to enhance anti-mycobacterial immune functions in phagocytes upon *in vitro* Mtb infection, which was dependent on its ability to reduce total cellular cholesterol and increase lysosomal acidification (Crowle and Ross, 1989; Wheelwright et al., 2014). Although vitamin-A deficiency strongly predicts risk of incident TB among HHC of TB patients, pre-clinical trials of vitamin-A supplementation has not been promising, likely due to the complex metabolic route required for conversion of vitamin-A to the active ingredient, ATRA (Lawson et al., 2010; Visser et al., 2011). The therapeutic impact of ATRA on MDSC has been evaluated in a TB mouse model, demonstrating that MDSC ablation restores T-cell numbers, reduces Mtb burden and decrease lung pathology (Knaul et al., 2014). More recently it was also shown that ATRA augments autophagy of Mtb in human and murine alveolar macrophages(Coleman et al., 2018) Further experiments are however required to fully establish the impact of ATRA on MDSC function in TB patients when employed as adjunctive therapy.

## Targeting MDSC in non-tuburculous intracellular infections

In addition to their involvement in Mtb infections, MDSC have versatile roles in other intracellular pathogenic infections (Dorhoi and Du Plessis, 2018). Their effect during these infections are governed by the pathogen species and the course of infection. Targeting of MDSC in other intracellular infections could thus involve either the reduction or expansion of their suppressive capacity and/or frequency, depending on their beneficial or detrimental impact on disease outcome. For example, MDSC are increased in both clinical and experimental viral infections such as HIV, SIV, and LP-BM5, contributing to pathogen survival through TH1 immunosuppression as observed by the correlation with viral load and CD4 T-cell count (Gama et al., 2012; Vollbrecht et al., 2012; Garg and Spector, 2014; O'Connor et al., 2016). Similar detrimental effects have been reported for cytomegalovirus (CMV) infections (Garg et al., 2017), with MDSC impairing viral clearance (Daley-Bauer et al., 2012). MDSC accumulation has been reported also for various non-tuberculous intracellular bacterial infections. *Staphylococcus aureus* infections are sustained by MDSC promoting an immunosuppressive environment and impairing macrophage responsiveness (Heim et al., 2015; Tebartz et al., 2015), whereas MDSC frequencies in *Francisella tularensis* infection correlate with the extent of tissue pathology, loss of pulmonary function and host mortality (Periasamy et al., 2016). Above mentioned depletion strategies might thus be applicable to a broad range of intracellular bacterial infections, although the timing of administration might require careful consideration.

## Conclusion

Tumors escape immune attack by a variety of mechanisms, often complementary in their ability to induce immunosuppression. Molecular interventions targeting innate immune cell regulatory pathways are making great advances in the immune-oncology field. Immune based cancer therapies are now also being recognized for their ability to potentiate anti-pathogen immunity when used in combination with classical treatment approaches. As for cancer, TB is described as a chronic inflammatory disease, characterized by a dysregulated immune profile. Data from cancer research suggest that MDSC can also be disarmed at pre-determined time points to redefine the outcome of disease. Therapeutic targeting of regulatory myeloid cells, such as MDSC and their molecular drivers, are, therefore, considered to be an exciting new strategy to help ameliorate TB via more effective and less-toxic strategies. Nevertheless, amongst the several approaches of TB HDT being sought, targeting of MDSC have not been explored, or their mechanistic involvement in the success of selected HDT, not appreciated. There is, however, a pressing need to study key signaling pathways and intermediates involved in the induction and function of regulatory myeloid cells, to allow pre-clinical screening of re-purposed drugs showing promise in oncology trials. For instance, the impact of metabolic pathways on MDSC function has only recently been recognized and requires investigation in the TB context. It will also be important to identify markers specific to MDSC, in particular, the predominant monocytic subset, to advance identification and development of suitable MDSC-targeting TB immunotherapies. We propose that MDSC remain an under investigated regulatory myeloid cell population that holds great promise in the TB HDT field.

## Author contributions

GW and NDP conceptualized the manuscript. NDP, LAK, and VL designed and drafted the manuscript with input from GW.

### Conflict of interest statement

The authors declare that the research was conducted in the absence of any commercial or financial relationships that could be construed as a potential conflict of interest.
